# Ecological models for estimating breakpoints and prediction intervals

**DOI:** 10.1002/ece3.6955

**Published:** 2020-11-16

**Authors:** Jabed H. Tomal, Jan J. H. Ciborowski

**Affiliations:** ^1^ Department of Mathematics and Statistics Thompson Rivers University Kamloops BC Canada; ^2^ Department of Biological Sciences University of Windsor Windsor ON Canada; ^3^ Present address: Department of Biological Sciences University of Calgary Calgary AB Canada

**Keywords:** bootstrap, chlorophyll phosphorus, ecological breakpoints, environmental stress, loess, multimetric index, piecewise linear regression, quantile regression

## Abstract

1. The relationships between an environmental variable and an ecological response are usually estimated by models fitted through the conditional mean of the response given environmental stress. For example, nonparametric loess and parametric piecewise linear regression model (PLRM) are often used to represent simple to complex nonlinear relationships. In contrast, piecewise linear quantile regression models (PQRM) fitted across various quantiles of the response can reveal nonlinearities in its range of variation across the explanatory variable.
2. We assess the number and positions of candidate breakpoints using loess and compare the relative efficiencies of PLRM and PQRM to quantitatively determine the breakpoints' location and precision. We propose a nonparametric method to generate bootstrap confidence intervals for breakpoints using PQRM and prediction bands for loess and PQRM. We illustrated the applications using data from two aquatic studies suspected to exhibit multiple environmental breakpoints: relating a fish multimetric index of community health (MMI) to agricultural activity in wetlands' adjacent drainage basins; and relating cyanobacterial biomass to total phosphorus concentration in Canadian lakes.
3. Two statistically significant breakpoints were detected in each dataset, demarcating boundaries of three linear segments, each with markedly different slopes. PQRM generated less biased, more accurate, and narrower confidence intervals for the breakpoints and narrower prediction bands than PLRM, especially for small samples and large error variability. In both applications, the relationship between the response and environmental variables was weak/nonsignificant below the lower threshold, strong through the midrange of the environmental gradient, and weak/nonsignificant beyond the upper threshold.
4. We describe several advantages of PQRM over PLRM in characterizing environmental relationships where the scatter of points represents natural environmental variation rather than measurement error. The proposed methodology will be useful for detecting multiple breakpoints in ecological applications where the limits of variation are as important as the conditional mean of a function.

## Graphical abstract

Ecological models were proposed and compared to assess the number and positions of candidate breakpoints. The methods were illustrated on applications using datasets from two aquatic studies suspected to exhibit multiple environmental breakpoints: relating a fish multimetric index of community health to agricultural activity in wetlands' adjacent drainage basins; and relating cyanobacterial biomass to total phosphorus concentration in Canadian lakes. Two statistically significant breakpoints were detected in each dataset, demarcating boundaries of three linear segments, each with markedly different slopes.

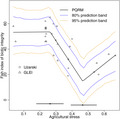

## 1 INTRODUCTION

“Biotic integrity” is defined as the “ability of a habitat to support and maintain a balanced, integrated, adaptive assemblage of organisms having a composition, diversity, and function comparable to that of a natural habitat (Frey, 1977).'' Maintaining biotic integrity of aquatic habitat is one of the primary objectives set forth by the US Clean Water Act of 1972 and by the European Union Water Framework Directive (EU WFD, 2000). Aggressive human activities and rapid urbanization leave only a few areas, if any, that can be considered as “natural habitat.” Instead, comparisons are commonly made to reference locations that are subject to a minimum level of anthropogenic stress (Stoddard et al., 2006). Questions of identifying the degree of disturbance at which biological changes, from reference to nonreference condition, occur across a stress gradient have long been an important consideration (Karr & Chu, 1998; Qian & Miltner, 2015). An ecological threshold (“numerical criterion”) is a point of abrupt change of the response variable of an ecological attribute (such as an index of ecological condition) relative to a measure of habitat, such as human‐induced disturbance affecting natural habitat (Fahrig, 2001). Such values can serve as guidelines for the protection of environmental condition of sites, and as theoretical conservation or restoration targets (Johnson, 2013; Larned & Schallenberg, 2019).

When the goals are to apply a precautionary principle to environmental management, the measures of biological response should be based on the limits of the relationships of the response given environmental condition (Cade et al., 1999). For example, Karanth et al. (2004) generated prediction bands for tiger densities as a function of their prey densities using standard centrality assumptions for the response variable. In this paper, we describe a method of generating bootstrap prediction bands for a biological response given environmental condition that is applicable to any ecological model without requiring constraining distributional assumptions for the error term.

Methods of identifying environmental disturbance thresholds have been a topic of considerable research. Regression trees (Bunea et al., 1999) and various parametric, nonparametric and Bayesian approaches have been applied to identifying the location of change points in the environmental stress‐biological condition relationship (Brenden et al., 2008; Dodds et al., 2010; Qian et al., 2003). The regression tree approach of Bunea et al. (1999), the nonparametric approach of Qian et al. (2003), and the nonparametric deviance reduction method of Brenden et al. (2008) are all similar and based on the concept of classification and regression tree (CART) models. Bayesian change‐point models evaluated by Qian et al. (2003) and Brenden et al. (2008) are the same model. The models reviewed by Brenden et al. (2008) are designed to detect one change point, which is discontinuous at the inflection point, and based on the concept of the nonparametric CART model.

Piecewise linear regression model (PLRM) is often used to estimate the location of environmental thresholds—typically a single breakpoint (Ficetola & Denoël, 2009; Shea & Vecchione, 2002; Toms & Lesperance, 2003; Toms & Villard, 2015). Such models may appear to be limited in two perspectives. Firstly, these models often use aggregated community metrics and consider that the regression relationship is based upon the association of two aggregated metrics each drawn from a single population of values. Yet, the sample data are often collected from many different systems representing multiple taxa, each with differing component properties. And the PLRM, which goes through the conditional mean of the metric representing biological response given the other metric representing environmental condition, may not capture the discontinuity in association present in other quantiles of the conditional distribution. This may make it difficult to identify the precise location of the change points (King & Baker, 2011). Secondly, a limiting factor (the least available factor among all factors in the aggregated metrics (Thomson et al., 1996)) may induce an unequal variance pattern in the biological response via interactions among the constituent factors incorporated in the aggregated metric representing environmental condition, and this can alter the relationships near the center of the conditional distribution of the biological response given environmental condition (Cade et al., 1999). Such limiting factors may also cause wedge‐shaped relationships in the conditional distribution of the biological response with respect to environmental condition; as a result, the relationships at the edges of the conditional distribution might appear to be more important than the relationship in the center of the distribution (Cade & Noon, 2003; Cade et al., 1999).

Quantile regression has the potential to accommodate these limitations in that it can estimate relationships between variables defined through different quantiles of the conditional distribution of the response variable. As a result, quantile regression models provide a more complete view of the possible relationships between variables than central tendency models (Cade & Noon, 2003). Cade and Noon (2003) gave a general overview of ecological applications of quantile regression, and discussed linear and nonlinear models with both homogeneous and heterogeneous error variances. Using a large simulation study, Cade et al. (2005) showed that the quantile regression model can reveal hidden bias and uncertainty in habitat models. They also showed that the parameters measured at upper (τ>0.5) and lower (τ<0.5) quantiles are less biased than the parameters defined at the mean of the conditional distribution of the response variable given the predictors in the presence of confounding variables. Austin (2007) reviewed the ecological applications of linear and nonlinear quantile regressions into species response models used in conservation. Bissinger et al. (2008) predicted marine phytoplankton maximum growth rates from temperature using a nonlinear quantile regression model. Planque and Buffaz (2008) used a linear quantile regression model to study fish recruitment‐environment relationships in marine ecology. Bryce et al. (2010) employed linear quantile regression to predict the maximum decline of vertebrate and macroinvertebrate assemblage responses against streambed sedimentation. Cade et al. (2005) used linear and nonlinear quantile regression models to reveal hidden bias and uncertainty in habitat models in ecology. Brenden et al. (2008) concluded that quantile regression was the most effective means of detecting a single disturbance threshold of the various approaches they investigated.

Nonparametric regression is another frequently used and effective means of studying simple to complex relationships between a biological response and an environmental stress variable. Trexler and Travis (1993) discussed the application of locally estimated scatterplot smoothing (loess) in ecology. Building on the recommendations of Toms and Lesperance (2003), we used loess to subjectively identify the number and positions of candidate ecological breakpoints along an environmental stressor gradient. We then complemented the use of the loess and the piecewise linear regression model (PLRM) approaches with a novel application of a piecewise linear quantile regression model (PQRM) to estimate the location of the environmental thresholds. We propose a method of quantile‐based bootstrap confidence interval (CI) for the environmental thresholds using the PQRM and compare estimates with the parametric CI of the breakpoints inferred using the PLRM.

We compared the performances of our methods using simulated data and illustrated the procedures by applying them to two datasets. Our objectives are threefold. The first objective is to display the shape of the relationship between a biological response and an environmental predictor variable. As per the second objective, we identify the locations and precision of the thresholds. Our third objective is to determine the prediction band for the biological response given environmental condition. We then discuss which method (PLRM versus PQRM) provides more precise estimates of environmental thresholds and the prediction bands; and whether PQRM provides more information about the relationships between variables than the PLRM.

## 2 MATERIALS AND METHODS

### 2.1 Loess and bootstrap prediction band

Let y and x be the biological response and environmental stress variables, respectively. The nonparametric locally estimated scatterplot smoothing (*loess*) model (Cleveland, 1979; Cleveland & Devlin, 1988) is defined as

(1) y=m(x;h)+ϵ,

where m(x;h) is the smoothed function of interest with smoothing parameter h and *ϵ* is an independent error term with mean 0 and standard deviation σ. Loess can capture both linear and nonlinear relationships between variables. Here, the goal of fitting loess is to approximate the number and positions of the breakpoints in a relationship by inspection. We used the R (R Core Team, 2020) statement loess to fit the model (Equation 1).

We generated a bootstrap (Efron & Tibshirani, 1994) prediction band for loess to provide a measure of the variability of the biological response given the environmental condition (Algorithm 1). The algorithm for generating the prediction band is obtained by adapting the methods of Hӓrdle and Bowman (1988) and Davison and Hinkley (1997).

The proposed algorithm assumes that the model is correctly specified and that the residuals are identically and independently distributed. However, the algorithm requires no distributional assumptions for the residuals. Importantly, it can be applied to any method by replacing m(x;h) by the desired model. In this algorithm, steps i–ii capture the sampling variability of the estimated model, and steps iii–iv capture the extra variability due to prediction.

Algorithm 1 Bootstrap resampling method to construct a nonparametric prediction band for the biological response given the predictor. The confidence level and number of bootstrap samples are represented by γ∈{0.80,0.95} and B, respectively.

**Table:** 

1. Fit a model $\hat{m}\left(x;h\right)$, and make prediction ${\hat{y}}_{i}=\hat{m}\left(x_{i};h\right)$ for i=1,2,⋯,n.
2. Calculate i th residual ${\hat{\epsilon }}_{i}=y_{i}‐{\hat{y}}_{i}$, and normalize the residuals as ${\tilde{\epsilon }}_{i}={\hat{\epsilon }}_{i}‐\frac{1}{n}\sum_{j=1}^{n}{\hat{\epsilon }}_{j}$ for i=1,2,⋯,n.
3. For bin1toB:
(i) Generate bootstrap residuals $\left\{\epsilon_{1}^{*},\epsilon_{2}^{*},\cdots ,\epsilon_{n}^{*}\right\}$ by sampling with replacement from $\left\{{\tilde{\epsilon }}_{1},{\tilde{\epsilon }}_{2},\cdots ,{\tilde{\epsilon }}_{n}\right\}$, and calculate bootstrap observations $y_{i}^{*}=\hat{m}\left(x_{i};h\right)+\epsilon_{i}^{*}$.
(ii) Fit a model ${\hat{m}}^{*}\left(x;h\right)$ using the bootstrapped observations $\left(x_{i},y_{i}^{*}\right)$, and calculate bootstrapped residuals $e_{i}^{*}=y_{i}^{*}‐{\hat{m}}^{*}\left(x_{i};h\right)$ for i=1,2,⋯,n.
(iii) Normalize the bootstrapped residuals ${\tilde{e}}_{i}^{*}=e_{i}^{*}‐\frac{1}{n}\sum_{j=1}^{n}e_{j}^{*}$ for i=1,2,⋯,n.
(iv) Sample residuals $\left\{e_{1}^{**},e_{2}^{**},\cdots ,e_{n}^{**}\right\}$ with replacement from the normalized bootstrapped residuals $\left\{{\tilde{e}}_{1}^{*},{\tilde{e}}_{2}^{*},\cdots ,{\tilde{e}}_{n}^{*}\right\}$, and calculate predicted residuals $e_{i}^{*p}=\hat{m}\left(x_{i};h\right)‐{\hat{m}}^{*}\left(x_{i};h\right)+e_{i}^{**}$ for i=1,2,⋯,n.
4. End For.
5. Calculate empirical quantiles $e_{i}^{*p}\left(\left(1‐\gamma \right)/2\right)$and $e_{i}^{*p}\left(\left(1+\gamma \right)/2\right)$of the predicted residuals across bootstrap resamples, and construct lower and upper limits of the prediction band $\left[{\hat{y}}_{i}+e_{i}^{*p}\left(\left(1‐\gamma \right)/2\right),{\hat{y}}_{i}+e_{i}^{*p}\left(\left(1+\gamma \right)/2\right)\right]$.

### 2.2 Piecewise linear regression model (PLRM)

A PLRM goes through the conditional mean of the response variable and connects two linear segments at each breakpoint. We define

$$y_{i}=m\left(x_{i};\beta ,\alpha \right)+\epsilon_{i}$$

to estimate two breakpoints incorporating three linear segments (Seber & Wild, 2003) as

(2) $$m\left(x_{i};\beta ,\alpha \right)=\left\{\begin{array}{ccc} \beta_{0}+\beta_{1}x_{i} & \mathrm{for} & x_{i}\leq \alpha_{1}\\ \beta_{0}+\beta_{1}x_{i}+\beta_{2}\left(x_{i}‐\alpha_{1}\right) & \mathrm{for} & \alpha_{1}<x_{i}\leq \alpha_{2}\\ \beta_{0}+\beta_{1}x_{i}+\beta_{2}\left(x_{i}‐\alpha_{1}\right)+\beta_{3}\left(x_{i}‐\alpha_{2}\right) & \mathrm{for} & x_{i}>\alpha_{2} \end{array}\right)$$

where $y_{i}$ and $x_{i}$ are the values for the i th response and predictor variables, respectively, and $\alpha_{1}$ and $\alpha_{2}$ are the breakpoints. Here, $\beta ={\left(\beta_{0},\beta_{1},\beta_{2},\beta_{3}\right)}^{T}$ represents the vector of regression coefficients and $\alpha ={\left(\alpha_{1},\alpha_{2}\right)}^{T}$ represents the vector of breakpoints. This model assumes that the errors $\epsilon_{i}$ are iid normal random variable with mean zero and standard deviation σ. The parameters (β,α,σ) are estimated using a nonlinear least squares method. We fitted PLRM (Equation 2) using the R package segmented (Muggeo, 2015). To run the nonlinear least squares method, we supplied the initial parameter values estimated from the fitted loess model.

The prediction band for the conditional distribution of the biological response given environmental condition is obtained by subtracting the margin of errors from the predicted values. The margin of errors is calculated by multiplying appropriate t values by the standard errors of prediction.

### 2.3 Piecewise linear quantile regression model (PQRM)

Quantile regression models (Cade & Noon, 2003; Koenker, 2005) are defined through the quantiles of the conditional distribution of the biological response variable. Such models allow one to evaluate relationships among variables through the conditional median of the biological response, as well as the full range of other conditional quantile functions. By supplementing the classical regression model, which is defined at the conditional mean, quantile regression models provide a more complete statistical analysis of the relationships among ecological variables (Mosteller & Tukey, 1977). The PQRM, which is defined at conditional quantiles, provides much richer information in terms of estimating a relationship and breakpoints than the PLRM, which is defined at the conditional mean.

Let $m_{\tau }\left(x;\beta_{\tau },\alpha_{\tau }\right)$ be the τ th quantile of the conditional distribution of the ecological response given environmental condition as.

$$y_{\tau }=m_{\tau }\left(x;\beta_{\tau },\alpha_{\tau }\right)+\epsilon_{\tau }.$$

Then the PQRM with two breakpoints is defined as:

(3) $$m_{\tau }\left(x_{i};\beta_{\tau },\alpha_{\tau }\right)=\left\{\begin{array}{ccc} \beta_{0\tau }+\beta_{1\tau }x_{i} & \mathrm{for} & x_{i}\leq \alpha_{1\tau }\\ \beta_{0\tau }+\beta_{1\tau }x_{i}+\beta_{2\tau }\left(x_{i}‐\alpha_{1\tau }\right) & \mathrm{for} & \alpha_{1\tau }<x_{i}\leq \alpha_{2\tau }\\ \beta_{0\tau }+\beta_{1\tau }x_{i}+\beta_{2\tau }\left(x_{i}‐\alpha_{1\tau }\right)+\beta_{3\tau }\left(x_{i}‐\alpha_{2\tau }\right) & \mathrm{for} & x_{i}>\alpha_{2\tau } \end{array}\right)$$

where $\alpha_{1\tau }$and $\alpha_{2\tau }$are the first and second breakpoints, respectively, defined at the τth quantile of the conditional distribution. Here, $\beta_{\tau }={\left(\beta_{0\tau },\beta_{1\tau },\beta_{2\tau },\beta_{3\tau }\right)}^{T}$ represents the vector of regression coefficients and $\alpha_{\tau }={\left(\alpha_{1\tau },\alpha_{2\tau }\right)}^{T}$ represents the vector of breakpoints defined at the τth quantile. The advantage of quantile regression is that there is no restriction for any distribution of the error term $\epsilon_{\tau }$. We used the statement nlrq of the R package quantreg (Koenker et al., 2018) to fit PQRM where the initial values of the parameters are supplied from the fitted loess.

Following Feng et al. (2011), we used wild bootstrap residuals to fit multiple PQRMs defined at the median to calculate the confidence interval (CI) for the breakpoints. The bootstrap CIs for the breakpoints of the PQRM at the median are obtained using Algorithm 2. In this algorithm, f is the kernel density function of the distribution of the error term $\epsilon_{i}$, $h_{i}=x_{i}^{2}/\sum_{j}x_{j}^{2}$ and $\psi_{\tau }\left(\epsilon_{i}\right)=\tau ‐I\left(\epsilon_{i}<0\right)$.

Algorithm 2 Bootstrap confidence intervals for the breakpoints using piecewise linear quantile regression model (PQRM). The confidence level and number of bootstrap samples are denoted by γ∈{0.95} and B, respectively.

**Table:** 

1. Fit a PQRM $\hat{m}\left(x;\beta ,\alpha \right)$, and make prediction ${\hat{y}}_{i}=\hat{m}\left(x_{i};\beta ,\alpha \right)$ for i=1,2,⋯,n.
2. Calculate ith residual ${\hat{\epsilon }}_{i}=y_{i}‐{\hat{y}}_{i}$, and normalize as ${\tilde{\epsilon }}_{i}={\hat{\epsilon }}_{i}‐{\left\{\hat{f}\left(0\right)\right\}}^{‐1}h_{i}\psi_{\tau }\left({\hat{\epsilon }}_{i}\right)$ for i=1,2,⋯,n.
3. For bin1toB:
(i) Generate the weights $w_{i}$ from the two‐point mass distribution
$w=\left\{\begin{array}{ccc} 2\left(1‐\tau \right) & \text{with probability} & 1‐\tau \\ ‐2\tau & \text{with probability} & \tau \end{array}\right)$
and calculate $\epsilon_{i}^{*}=w_{i}\left|{\tilde{\epsilon }}_{i}\right|$ and bootstrap observations $y_{i}^{*}=\hat{m}\left(x_{i};\beta ,\alpha \right)+\epsilon_{i}^{*}$.
(ii) Fit a PQRM $\hat{m}\left(x_{i};\beta^{*},\alpha^{*}\right)$ using the bootstrapped observations $\left(x_{i},y_{i}^{*}\right)$, and calculate the breakpoints $\left({\hat{\alpha }}_{1}^{*},{\hat{\alpha }}_{2}^{*}\right)$.
4. End For.
5. Calculate empirical quantiles to construct the lower and upper limits of the confidence intervals for $\alpha_{1}$as $\left[{\hat{\alpha }}_{1}^{*}\left(\left(1‐\gamma \right)/2\right),{\hat{\alpha }}_{1}^{*}\left(\left(1+\gamma \right)/2\right)\right]$and for $\alpha_{2}$as $\left[{\hat{\alpha }}_{2}^{*}\left(\left(1‐\gamma \right)/2\right),{\hat{\alpha }}_{2}^{*}\left(\left(1+\gamma \right)/2\right)\right]$.

## 3 APPLICATIONS

### 3.1 Relating a wetland fish multimetric index (MMI) to variation in agricultural stress among Laurentian Great Lakes coastal wetlands

The first application relates to estimating threshold effects of a measure of agricultural activity in watersheds draining into the Laurentian Great Lakes on scores of a multimetric index of community composition of fishes in bordering coastal wetlands (Bhagat et al., 2007). Runoff associated with agriculture is a major source of human‐induced disturbance affecting natural habitat loss for fishes (Brazner & Beals, 1997; Crosbie & Chow‐Fraser, 1999). Danz et al. (2005) derived a composite agricultural stress index (AG) to characterize the risk of degradation of natural habitat using GIS‐based data. We rescaled the AG (a PCA score) to a 0–1 range with larger numbers reflecting more extensive agricultural activities. The measure of biological condition is a wetland fish multimetric index (MMI), a measure representing the inferred health of the fish assemblage in an ecoregion or watershed. Uzarski et al. (2005) developed and Bhagat et al. (2007) validated the fish multimetric index by assessing fish assemblages in stands of bulrush (*Schoenoplectus*, spp) in 30 coastal wetland distributed across the US Great Lakes coast (Table A1). Scores vary from 0 to 100, with larger scores representing greater ecological health of the fish assemblage. Traditionally, MMI scores falling in the lowest and highest quintiles are classified as “degraded” and “excellent” conditions, respectively. Bhagat et al. (2007) observed a statistically significant negative linear association between fish IBI and AG scores, but suggested the presence of threshold responses. They did not quantitatively test for the presence of breakpoints.

### 3.2 Relating cyanobacteria biomass to total phosphorus concentrations among lakes

The second application relates to identifying putative threshold effects of total phosphorus (TP) on the risk of development of harmful algal blooms (dominated by toxigenic Cyanobacteria) in lakes (Beaulieu et al., 2014; Downing et al., 2001; Watson et al., 1992). TP is a limiting nutrient whose loads to lakes and rivers reflect contributions of sewage from urban centers, agricultural runoff, and other manifestations of human activity (Qian et al., 2003; Reynolds & Walsby, 1975). Cyanobacteria biomass per unit volume (CB) is a standard index of concentration, and often used as a proxy for the risk of toxicity of harmful algal blooms. Cyanobacteria blooms are manifestations of eutrophication whose prevalence is increasing globally (Bullerjahn et al., 2016). CB harbors compounds that can be acutely toxic (Campos & Vasconcelos, 2010; Roegner et al., 2014) and that are linked to diseases such as carcinoma (Labine & Minuk, 2009; Lone et al., 2015). Thus, CB is directly related to risks to human and animal health (Downing et al., 2001; Svendsen et al., 2018).

Opinion on the shape of the relationship between TP and CB is varied. TP is arguably one of the top single predictors of CB (Chlorophyll a), and empirically derived linear models are widely used in lake management (Beaulieu et al., 2014; Dillon & Rigler, 1974, 1975; Stow & Cha, 2013). However, sigmoidal relationships between TP and CB are also well documented (Chow‐Fraser et al., 1994; Downing et al., 2001; Filstrup et al., 2014; Watson et al., 1992, 1997). Beaulieu et al. (2014) used data (Table A1) provided by the Ministries of the Environment of Alberta (43 lakes), British Columbia (10 lakes), and Ontario (97 lakes) relating to CB (μg/L) and TP (μg/L) concentrations. Using linear regression, nonlinear regression, and mixed‐effects models, they concluded that linear models better explained the data pattern than nonlinear approaches. Yet, scatterplots appear to indicate discontinuities in the TP‐CB relationship.

### 3.3 Simulation: Evaluating effects of sample size and precision

For the simulation, we generated data from the following model.

$$y_{i}=\beta_{0}+\beta_{1}x_{i}+\beta_{2}x_{i}I\left(x_{i}‐\alpha_{1}\right)+\beta_{3}x_{i}I\left(x_{i}‐\alpha_{2}\right)+\epsilon_{i}^{*}$$

such that $\epsilon_{i}^{*}={\left[|x_{i}‐\gamma_{1}|+\gamma_{2}\right]}^{‐1}\epsilon_{i}$, where $\epsilon_{i}∼t$‐distribution with df degrees of freedom. It is a piecewise linear regression model with varying error variances and heavy‐tailed t‐distribution. To assess the accuracy of each method as compared to known parameters, we selected the following values, based upon the estimates derived from the actual Fish MMI and agricultural stress data: $\beta_{0}=51.92$, $\beta_{1}=4.41$, $\beta_{2}=‐166.14$, $\beta_{3}=274.00$, $\alpha_{1}=0.26$, and $\alpha_{2}=0.49$. We varied x variable values from the smallest AG values of 0.0351 to the largest AG values of 0.6698. We further considered $\gamma_{1}$ to be 0.35 and $\gamma_{2}$ to be 0.10. This set‐up allows larger error variances to the values of x around 0.35 than the edges. Also, the simulated data values near the first breakpoint $\alpha_{1}$ are more variable than the simulated data values near the second breakpoint $\alpha_{2}$. We then evaluated the relative performance of each regression method by creating scenarios of sets of 100 simulated datasets for each of the 6 combinations of sample size (n=30 and 150) and error degrees of freedom (df=10, 15, and 20). For each dataset, a total of 1,000 bootstrap samples were generated from which to estimate the confidence intervals and prediction bands. We then calculated the bias, variance, and mean‐squared error (*MSE*) of the point estimates, and coverage and width for the confidence intervals of the two breakpoints. For the prediction bands of loess, PLRM, and PQRM, we calculated the mean areas under the curve with their standard errors as a function of sample size and error degrees of freedom.

## 4 RESULTS

### 4.1 Simulation results

We first present the results in terms of bias, variance, mean‐squared error (*MSE*), coverage, and width for the confidence intervals of the breakpoints identified by the PLRM and PQRM (Table 1). The biases, variances, and mean‐squared errors of the point estimates of the breakpoints are smaller for PQRM than for PLRM especially when the sample sizes and degrees of freedoms are small. The differences between the metrics (bias, variance, and *MSE*) for PLRM and PQRM become smaller as the sample sizes and degrees of freedoms grow larger. However, estimates for the first and second breakpoints are positively and negatively biased, respectively. For the first breakpoint, around which the generated data were more variable, the coverage for PQRM is larger than for PLRM. For the second breakpoint, around which the generated data were less variable, the coverage for PQRM is smaller than the coverage for PLRM. For the first breakpoint, the width of the confidence interval is smaller for PQRM than for PLRM especially for small samples. For the second breakpoint, the width of the confidence interval is smaller for PQRM than for PLRM for both small and large samples.

**Table 1:** Biases, variances (Var), and mean‐squared error (*MSE*) for the point estimates of the thresholds using piecewise linear regression model (PLRM) and piecewise linear quantile regression model (PQRM) under two sample sizes and three error degrees of freedoms. The coverage and width of the confidence intervals of the thresholds are also provided

Thresholds	Metrics	Methods	Sample Sizes (n)					
			30			150		
			df			df		
			10	15	20	10	15	20
$\alpha_{1}$	Bias	PLRM	0.0168	0.0148	0.0140	0.0039	0.0037	0.0043
		PQRM	0.0085	0.0078	0.0075	0.0019	0.0021	0.0037
	Var	PLRM	0.0032	0.0027	0.0026	0.0005	0.0004	0.0004
		PQRM	0.0016	0.0013	0.0013	0.0003	0.0004	0.0004
	*MSE*	PLRM	0.0035	0.0029	0.0028	0.0005	0.0004	0.0004
		PQRM	0.0016	0.0013	0.0013	0.0004	0.0004	0.0004
	Coverage	PLRM	0.6900	0.7000	0.7100	0.7900	0.7800	0.7800
		PQRM	0.7200	0.7500	0.7400	0.8200	0.7900	0.8000
	Width	PLRM	0.0999	0.0985	0.0997	0.0489	0.0470	0.0462
		PQRM	0.0913	0.0924	0.0898	0.0587	0.0579	0.0581
$\alpha_{2}$	Bias	PLRM	−0.0135	−0.0139	−0.0119	−0.0017	−0.0013	−0.0013
		PQRM	−0.0029	−0.0031	−0.0034	−0.0015	−0.0010	−0.0013
	Var	PLRM	0.0011	0.0012	0.0010	0.0001	0.0001	0.0001
		PQRM	0.0003	0.0003	0.0003	0.0001	0.0001	0.0001
	*MSE*	PLRM	0.0013	0.0014	0.0011	0.0001	0.0001	0.0001
		PQRM	0.0003	0.0003	0.0004	0.0001	0.0001	0.0001
	Coverage	PLRM	0.7900	0.8000	0.8100	0.9200	0.9100	0.9300
		PQRM	0.7500	0.7200	0.7200	0.8800	0.8800	0.9000
	Width	PLRM	0.0673	0.0656	0.0655	0.0317	0.0304	0.0298
		PQRM	0.0486	0.0485	0.0484	0.0284	0.0281	0.0278

The mean area within the prediction bands (AWC) and the standard errors against sample size (n) and error degrees of freedom (df) are shown for loess, PLRM, and PQRM (Table 2). For the 80% prediction band, the smallest area is from PQRM followed by loess and PLRM irrespective of whether sample sizes were small or large. For the 95% prediction band, the smallest area is from loess followed by PQRM and PLRM for small sample sizes (n=30). For the large sample (n=150), the smallest area is from PLRM followed by PQRM and loess.

**Table 2:** Average areas under the curve (with standard error) of the prediction bands (PB) for Loess, PLRM, and PQRM under two sample sizes and three error degrees of freedoms scenarios

PB	Methods	Sample Sizes (n)					
		30			150		
		df			df		
		10	15	20	10	15	20
80%	Loess	7.88 (0.14)	7.70 (0.13)	7.56 (0.12)	8.19 (0.06)	7.98 (0.06)	7.88 (0.06)
	PLRM	9.59 (0.20)	9.29 (0.18)	9.17 (0.18)	9.11 (0.09)	8.71 (0.08)	8.55 (0.08)
	PQRM	6.96 (0.13)	6.81 (0.13)	6.71 (0.12)	7.62 (0.07)	7.42 (0.06)	7.33 (0.06)
95%	Loess	13.29 (0.32)	12.90 (0.29)	12.71 (0.28)	14.65 (0.18)	14.04 (0.17)	13.78 (0.15)
	PLRM	15.02 (0.31)	14.55 (0.28)	14.36 (0.28)	13.99 (0.14)	13.38 (0.13)	13.12 (0.12)
	PQRM	14.29 (0.37)	13.91 (0.35)	13.65 (0.34)	14.47 (0.17)	13.87 (0.16)	13.63 (0.16)

In a nutshell, the performance of PQRM is less biased and more accurate than the PLRM in situations with small samples and large error variability reflected by small degrees of freedom.

### 4.2 Relationships between fish multimetric index and agricultural stress

#### 4.2.1 Loess and bootstrap prediction band

**Figure 1:**
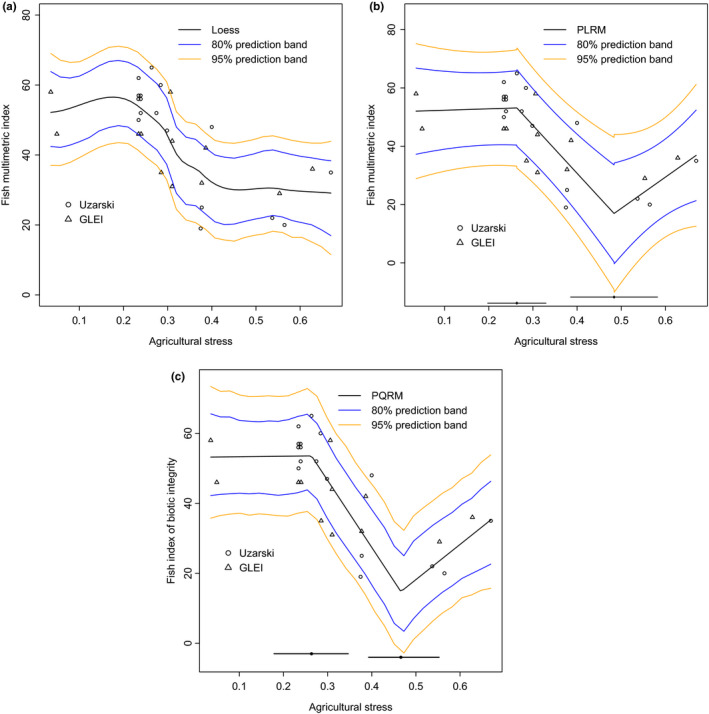
Fish MMI versus AG. Panel (a) shows fitted loess along with 80% and 95% prediction bands with surface areas 12.089 and 17.730 square units, respectively. Panel (b) shows fitted PLRM along with 80% and 95% prediction bands with surface areas of 17.240 and 27.000 square units, respectively. Panel (c) shows fitted PQRM along with 80% and 95% prediction bands with surface areas 13.668 and 22.078 square units, respectively. The two solid lines along the horizontal axis in panels (b) and (c) are the 95% CIs for the thresholds

The relationship between Fish MMI and AG was negative (Figure 1a). The loess suggests the possible presence of two breakpoints at AG values about 0.22 and 0.45. MMI score was independent of AG stress when AG scores were below 0.22, decreased sharply between stress values of 0.22 and 0.45, and reached its minimum at AG stress values over 0.45.

The bootstrap prediction bands for the Fish MMI provided an idea of the location of possible thresholds and uncertainty of the range of the Fish MMI response variable (Figure 1a). The 80% and 95% prediction bands covered surface areas of 12.089 and 17.730 square units, respectively (Table A9). For AG of 0.210 (the position below the first threshold), the predicted Fish MMI was 55.678 with 80% and 95% prediction intervals of (47.968, 66.805) and (43.381, 70.874), respectively. For agricultural stress 0.517 (the position above the second threshold), the predicted Fish MMI is 30.514 with 80% and 95% prediction intervals of (22.226, 41.185) and (17.667, 45.345), respectively. The upper limit of fish MMI of 41.185 using the 80% prediction band at AG value of 0.517 is lower than the lower limit of fish MMI of 47.968 using the 80% prediction band at AG value of 0.21.

#### 4.2.2 Piecewise linear regression model

The PLRM identified two breakpoints at AG scores of 0.263 and 0.488, respectively (Table A2), broadly corresponding to the location of inflection points identified by the loess model. There is no overlap between the CIs (0.196, 0.331) and (0.391, 0.585) for the two breakpoints. Thus, the two breakpoints are significantly different.

The slopes $\beta_{1}$ and $\beta_{1}+\beta_{2}+\beta_{3}$ of the first and third segments of PLRM were not significantly different from zero (t=0.122, p=0.904; t=1.392, p=0.177, respectively). Thus, Fish MMI scores were independent of AG stress at low levels of stress, up to 0.263, and at high‐stress levels exceeding 0.488. MMI score decreased significantly with increasing AG stress between the breakpoints 0.263 and 0.488 (t=3.009, p=0.006).

The 80% and 95% prediction bands for the Fish MMI covered surface areas of 17.240 and 27.000, respectively (Figure 1b). The PLRM prediction bands were substantially wider than those generated from loess, reflecting the large residual standard deviation $\hat{\sigma }=9.055$ (relative to standard deviation of 13.200 for Fish MMI) and the loss of degrees of freedom associated with estimating 6 parameters. For AG of 0.210, the predicted Fish MMI is 52.850 with 80% and 95% prediction intervals of (40.425, 65.275) and (33.391, 72.309), respectively. For AG value of 0.517, the predicted Fish MMI is 19.956 with 80% and 95% prediction intervals of (4.697, 35.215) and (−3.941, 43.853), respectively. The upper limit of fish MMI of 35.215 using the 80% prediction band at AG value of 0.517 (a value above the second threshold) is lower than the lower limit of fish MMI of 40.425 using the 80% prediction band at AG value of 0.21 (a value below the first threshold).

#### 4.2.3 Piecewise linear quantile regression model

The PQRM defined at the median (τ=0.50) of the conditional distribution of Fish MMI scores as a function of AG stress estimated the location of two significantly different breakpoints: ${\hat{\alpha }}_{1\tau }=0.264$ (CI of (0.179, 0.347); Table A3) and ${\hat{\alpha }}_{2\tau }=0.466$ (CI of (0.393, 0.553)). The Fish MMI remains flat (${\hat{\beta }}_{1\tau }=1.447$ with 95% CI (−41.363, 92.828)) with respect to AG scores up to 0.264, decreases sharply (${\hat{\beta }}_{1\tau }+{\hat{\beta }}_{2\tau }=‐191.537$ with 95% CI (−410.675, −107.144)) against AG from 0.264 to 0.466. The results estimate that the Fish MMI score increases slowly with increasing AG scores greater than 0.466 with slope ${\hat{\beta }}_{1\tau }+{\hat{\beta }}_{2\tau }+{\hat{\beta }}_{3\tau }=100.594$ and 95% confidence interval (28.830, 193.894). We believe that this counterintuitive result of apparent rising trend in MMI scores in the third segment of the model for AG scores greater than 0.466 is an artifact of the sparse data especially in the vicinity of the estimated breakpoint and small sample size.

Algorithm 1 was used to obtain the prediction bands for the Fish MMI defined at the median (Figure 1c). The 80% and 95% confidence bands covered surface areas of 13.668 and 22.078, respectively. As in the worst‐case simulation scenario of small n and df, the prediction bands for PQRM were narrower than those for PLRM. For AG of 0.210, the predicted Fish MMI is 53.472 with 80% and 95% prediction intervals of (42.580, 63.882) and (36.374, 70.495), respectively. For AG of 0.517, the predicted Fish MMI is 19.856 with 80% and 95% prediction intervals of (9.820, 31.537) and (4.141, 38.697), respectively. The upper limit of fish MMI of 31.537 using the 80% prediction band at AG value of 0.517 (a value above the second threshold) is lower than the lower limit of fish MMI of 42.580 using the 80% prediction band at AG value of 0.21 (a value below the first threshold).

The estimates of $\alpha_{1\tau }$ varied from 0.233 to 0.284 and $\alpha_{2\tau }$ varied from 0.448 to 0.564 across the conditional quantiles of the distribution of Fish MMI against AG (Table A4). Also, the estimates of $\beta_{1\tau }$ varied from −10.076 to 31.579, $\beta_{1\tau }+\beta_{2\tau }$ varied from −223.464 to −106.214, and $\beta_{1\tau }+\beta_{2\tau }+\beta_{3\tau }$ varied from 62.053 to 153.677.

### 4.3 Relationship between cyanobacteria biomass and total phosphorus

#### 4.3.1 Loess and bootstrap prediction band

**Figure 2:**
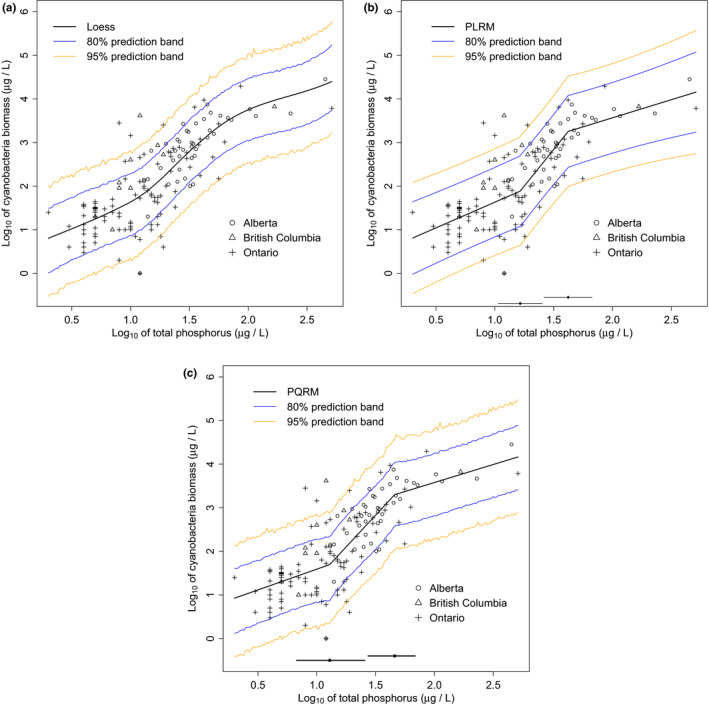
log of CB versus log of TP. The Panel (a) displays fitted loess along with 80% and 95% prediction bands with surface areas 3.445 and 5.948 square units, respectively. Panel (b) displays the fitted PLRM, 80% and 95% prediction bands with surface areas of 3.956 and 6.073 square units, respectively. Panel (c) represents fitted PQRM along with 80% and 95% prediction bands with surface areas 3.485 and 6.099 square units, respectively. The two solid lines along the horizontal axis in panels (b) and (c) are the marginal 95% CIs for the thresholds

The fitted loess and the prediction bands indicated that there was a positive relationship between $log_{10}\left(\mathrm{CB}\right)$ and $log_{10}\left(\mathrm{TP}\right)$ (Figure 2a). Discontinuities in the trend line suggested that there were two candidate breakpoints, one at around log(TP) of 1.20 (15.85 μg/L) and the other at around log(TP) of 1.70 (50.12 μg/L). CB increased steadily as function of log(TP) up to a value of 1.20, rose sharply between 1.20 and 1.70, and then rose more slowly at greater log(TP) concentrations. The 80% and 95% bootstrap prediction bands (which covered surface areas of 3.445 and 5.948, respectively) identified the potential range of CB of a given value of log(TP). For log(TP) of 1.012 (a point below the first threshold), the predicted log(CB) was 1.656 with 80% and 95% prediction intervals (0.879, 2.309) and (0.389, 2.730), respectively. For log(TP) of 2.015 (a point above the second threshold), the predicted log(CB) was 3.769 with 80% and 95% prediction intervals (3.052, 4.481) and (2.523, 4.959), respectively. The lower limit of log(CB) of 3.052 using the 80% prediction band at log(TP) of 2.015 is higher than the upper limit of log(CB) of 2.309 using the 80% prediction band for log(TP) of 1.012.

#### 4.3.2 Piecewise linear regression model

The PLRM identified two breakpoints at log(TP) of 1.212 (16.293 μg/L) and 1.624 (42.073 μg/L) with 95% CIs (1.026, 1.399) and (1.418, 1.830), respectively (Table A5). The breakpoints were significantly different as there was no overlap between the intervals.

The trends of CB against TP estimated by the PLRM (Figure 2b) were consistent in relative magnitude with the patterns subjectively described by the loess. Values for regression coefficients in the first and second segments (but not the third segment) of the regression lines were significantly greater than zero.

The 80% and 95% prediction bands covered surface areas of 3.956 and 6.073 square units, respectively. The PLRM prediction bands were substantially wider than those generated from loess. This is due to large $\hat{\sigma }=0.614$ (comparing to the standard deviation of 0.990 for log(CB)) and the loss of degrees of freedom for the estimation of 6 parameters. For log(TP) of 1.012 (a point below the first threshold), the predicted log(CB) was 1.645 with 80% and 95% prediction intervals (0.847, 2.443) and (0.420, 2.870), respectively. For log(TP) of 2.015 (a point above the second threshold), the predicted log(CB) was 3.580 with 80% and 95% prediction intervals (2.767, 4.392) and (2.332, 4.827), respectively. The lower limit of log(CB) of 2.767 using the 80% prediction band at log(TP) of 2.015 is higher than the upper limit of log(CB) of 2.443 using the 80% prediction band at log(TP) of 1.012.

#### 4.3.3 Piecewise linear quantile regression model

The PQRM defined at the median (τ=0.50) of the conditional distribution provided estimates of the two breakpoints ${\hat{\alpha }}_{1\tau }=1.110$ and ${\hat{\alpha }}_{2\tau }=1.662$ (Table A6) with 95% bootstrap CIs (0.829, 1.408) and (1.437, 1.836), respectively. The breakpoints were significantly different from each other as the CIs didn't overlap. The relationship was not statistically significant at the lowest concentrations of TP (${\hat{\beta }}_{1\tau }=0.952$ with 95% CI (−0.316, 1.816)). There was a strong positive relationship between the two variables for the middle segment (${\hat{\beta }}_{1\tau }+{\hat{\beta }}_{2\tau }=2.904$ with 95% CI (2.121, 5.373)). At values of log(TP) over 1.662, log(CB) then increased slowly (${\hat{\beta }}_{1\tau }+{\hat{\beta }}_{2\tau }+{\hat{\beta }}_{3\tau }=0.825$; with 95% CI (0.214, 1.337)). The estimated residual standard deviation was ${\hat{\sigma }}_{\tau }=0.618$.

Algorithm 1 was used to obtain prediction bands bounding the CB‐TP relationship (Figure 2c). The 80% and 95% prediction bands covered surface areas of 3.485 and 6.099, respectively, for the range of the CB values expected for a given TP concentration. For log(TP) of 1.012, the predicted log(CB) was 1.603 with 80% and 95% prediction intervals (0.835, 2.299) and (0.331, 2.784), respectively. For log(TP) of 2.015, the predicted log(CB) was 3.591 with 80% and 95% prediction intervals (2.827, 4.257) and (2.261, 4.780), respectively. The lower limit of log(CB) of 2.827 using the 80% prediction band at log(TP) of 2.015 (a point above the second threshold) is higher than the upper limit of log(CB) of 2.299 using the 80% prediction band at log(TP) of 1.012 (a point below the first threshold).

The estimates of $\alpha_{1\tau }$ varied from 1.066 to 1.230 and $\alpha_{2\tau }$ varied from 1.585 to 1.662 across the conditional quantiles of the distribution of CB against TP (Table A7). The estimates of $\beta_{1\tau }$, $\beta_{1\tau }+\beta_{2\tau }$, and $\beta_{1\tau }+\beta_{2\tau }+\beta_{3\tau }$ varied from 0.364 to 1.958, 2.679 to 4.414, and 0.647 to 1.269, respectively.

## 5 COMPARISONS

We examined which method provided the narrowest CIs of the breakpoints (Table A8). For the Fish MMI and AG data, PLRM generated a narrower interval for $\alpha_{1}$, and PQRM generated a narrower interval for $\alpha_{2}$. Similarly, for the CB versus TP data, PLRM generated a narrower interval for $\alpha_{1}$, and PQRM generated a narrower interval for $\alpha_{2}$.

We also compared the surface areas bounded by the prediction bands (Table A9). For the 80% prediction band of the Fish MMI versus AG data, the surface areas covered by loess, PLRM, and PQRM were 12.089, 17.240, and 13.668 square units, respectively. For the 95% prediction band, the surface areas covered by loess, PLRM and PQRM were 17.730, 27.000, and 22.078 square units, respectively. The smallest surface area was derived from loess followed by PQRM and PLRM. For the CB versus TP data, the surface areas of 80% prediction bands are 3.445, 3.956, and 3.485 square units, respectively. The surface areas of 95% prediction bands are 5.948, 6.073, and 6.099 square units, respectively. The smallest surface area was derived from loess. The surface areas from PLRM and PQRM are close to each other.

## 6 DISCUSSION AND CONCLUSIONS

### 6.1 Fish multimetric index versus agricultural stress

Loess, PLRM, and PQRM all identified 2 breakpoints in the same locations along the AG stress. Of the two methods from which CIs could be empirically calculated, the 95% CI of the PQRM was 124.44% and 82.47% as wide as those of the PLRM for the lower and upper thresholds, respectively. PQRM produced a narrower CI for the second breakpoint demarcating the sharp transition from second regime to the third.

The PLRM approach identified breakpoints that were significantly different from each other and represented marked discontinuities in the MMI‐AG relationship. Fish MMI scores were independent of AG stress range below 0.263, but were a significant negative function of increasing AG from 0.263 to 0.488. Fish MMI was also independent of AG at stress values over 0.488. Similar results were obtained from PQRM. Tests for the slope indicated that fish MMI score was a negative function of AG between the two stress thresholds. The upper limit of fish MMI using the 80% prediction band given AG score of 0.517 (a point above the second threshold) was lower than the lower limit of fish MMI using the 80% prediction band given AG score of 0.210 (a point below the first threshold).

The ecological and environmental management implications (Larned & Schallenberg, 2019) of this interpretation are significant. The results suggest that AG values less than 0.263 have no detectable influence on the fish assemblages, relative to the range of natural variation. In contrast, under high levels of AG (>0.488) management practices that slightly reduce agricultural effects are unlikely to improve fish assemblage condition as expressed in MMI scores. Agricultural changes to watersheds draining into coastal wetlands are only likely to influence fish community condition in wetlands with stress scores between the two breakpoints.

The loess prediction envelope was the smallest: The PQRM enveloped an area of 113.06% and 124.52% for the 80% and 95% prediction bands, respectively, of that produced by the loess. The PQRM produced the narrower prediction bands enveloping an area of 79.28% and 81.77% for the 80% and 95% prediction bands, respectively, than PLRM. These results of smaller prediction band using PQRM than PLRM match the simulation results of the worst‐case scenario of small sample size and large error variability.

PQRM revealed important information for the Fish MMI versus AG relationship confirming the presence of discontinuities (thresholds) in the stress‐response relationship that were qualitatively suggested by the original researchers (Bhagat et al., 2007). For example, the lines in the central segment of the model showed varying values of slopes across the conditional quantiles. For the lower quantiles (τ≤0.60) of the MMI versus AG relationship, the slope varied from −223.464 to −176.737 indicating large decline in MMI against AG, whereas for the upper quantiles (τ>0.60) the slope varied from −110.634 to −106.214 indicating smaller decline (less sensitivity) of fish MMI to AG. These additional results from PQRM reflect its strengths over PLRM, which models the relationship only through the conditional mean of the biological response against environmental stress. The additional slope estimates provided by the quantile regression analysis can guide restoration ecologists' expectations as to the likelihood that management practices that reduce agricultural stresses emanating from watersheds will alter fish community health (Johnson, 2013). If the biological variable is truly controlled by environmental stress, then management actions are likely to be most effective at locations where AG scores fall within the central segment of the stress range. Furthermore, Locations in which MMI scores correspond to lower quantiles (τ≤0.60) are likely to be more sensitive to management actions than locations whose fish assemblages have relatively high MMI scores for a particular conditional stress level (τ>0.60).

### 6.2 Cyanobacterial biomass versus total phosphorus

Loess, PLRM, and PQRM all identified breakpoints in the same locations along the log(TP) stress. Using PLRM, the tests for the presence of ecological breakpoints identified two statistically significant breakpoints, corresponding to log(TP) of 1.212 (16.293 μg/L) and 1.624 (42.073 μg/L), respectively. CB increased slowly for log(TP) concentrations below 1.212, sharply between 1.212 and 1.624, and slowly at higher log(TP) concentrations above 1.624. For the first and second thresholds, the 95% confidence intervals were narrower for PLRM and PQRM, respectively. Using PQRM, the slope in the first segment was not statistically significantly different from zero. The slopes of the second and third segments indicate a significantly positive relationship between CB and TP. However, both the range of variation in CB and the slope of the relationship is much steeper over the range of TP concentrations between 12.882 μg/L (log(TP)=1.110) and 45.920 μg/L (log(TP)=1.662). Below the lower threshold, CB is consistently less than 199.067 μg/L (log(CB)=2.299) at log(TP) of 1.012, whereas above the upper threshold, CB is predicted to be greater than 671.429 μg/L (log(CB)=2.827) at log(TP) of 2.015, ranging by a factor of at least *three* between the two observed TP concentrations (Figure 2c).

The area of the 80% prediction band estimated by the PQRM was narrower (88.09%) than the band estimated by PLRM. However, the 95% prediction bands for PQRM and PLRM were similar. The areas of the prediction bands estimated by PQRM were minimally wider than the band estimated by the loess. This is probably due to large sample size (n=150), and large residual standard deviations of $\hat{\sigma }=0.614$ and ${\hat{\sigma }}_{\tau }=0.618$ for PLRM and PQRM, respectively, compared to the standard deviation of 0.990 for the log(CB).

The information revealed by PQRM is very important for interpreting the CB versus TP relationship. For example, the linear lines in the second segment of the model show varying values of slopes across the conditional quantiles. For the lower quantiles (τ<0.50) of the CB versus TP relationship, the slope varies from 3.532 to 4.414 indicating a large increase in CB versus TP, whereas for the upper quantiles (τ≥0.50) the slope varies from 2.679 to 2.946 indicating a smaller increase (less sensitivity) in CB relative to TP. Quantification of these types of relationships across multiple quantiles of the conditional distribution of the biological response against environmental stress is not possible using the PLRM, which is defined only at the conditional mean of the distribution.

Biomass was interpreted to be a monotonically increasing function of TP by Dillon and Rigler (1974) and Beaulieu et al. (2014). But the identification of 3 significantly different segments of the relationship separated by breakpoints in the nutrient gradient supports the sigmoidal interpretation of the relationship (Filstrup et al., 2014; Watson et al., 1992). The management implications (Larned & Schallenberg, 2019) associated with applying single‐slope versus 3‐segmented interpretations of the relationship are significant. Use of a linear model to guide management implies that any alteration in TP concentration in a receiving water body can be expected to elicit a cyanobacterial response. In contrast, adherence to a sigmoidal model implies that there are points of inflection beyond which the two variables may behave independently, possibly obviating the need for the control of TP below a particular threshold concentration. Phosphorus has traditionally been regarded as the key nutrient limiting phytoplankton biomass in lakes (Schindler et al., 2008). However, Beaulieu et al. (2014) observed that CB values could be predicted equally well and with similar patterns from concentrations of Total Nitrogen (TN). Yet, a strong correlation between TP and TN, made it difficult for the authors to identify which of the two nutrients was the ultimate predictor of CB. The trisegmented relationship that we observed could reflect colimitation of these two nutrients (Müeller & Mitrovic, 2015). A strength of PQRM is that bivariate relationships can be modeled even when potential confounding factors add variation that reduces the signal to noise ratio in the center of the conditional distribution (Cade & Noon, 2003).

We have proposed methods for quantifying the positions and precision of breakpoints that are subjectively identified by empirical observations, justified by visual analysis of the relationships between a response variable and its predictor using nonparametric loess, which minimized potential observer biases. Loess, PLRM, and PQRM all identified breakpoints in the same stress locations, which were statistically significantly nonoverlapping. As a result, we rule out the possibility that these ecological thresholds are spurious (Daily et al., 2012). However, our observations are consistent with the findings of Daily et al. (2012) that greater precision in estimation is achieved with larger sample sizes and a higher frequency of observations across the environmental stress gradient.

**Figure A1:**
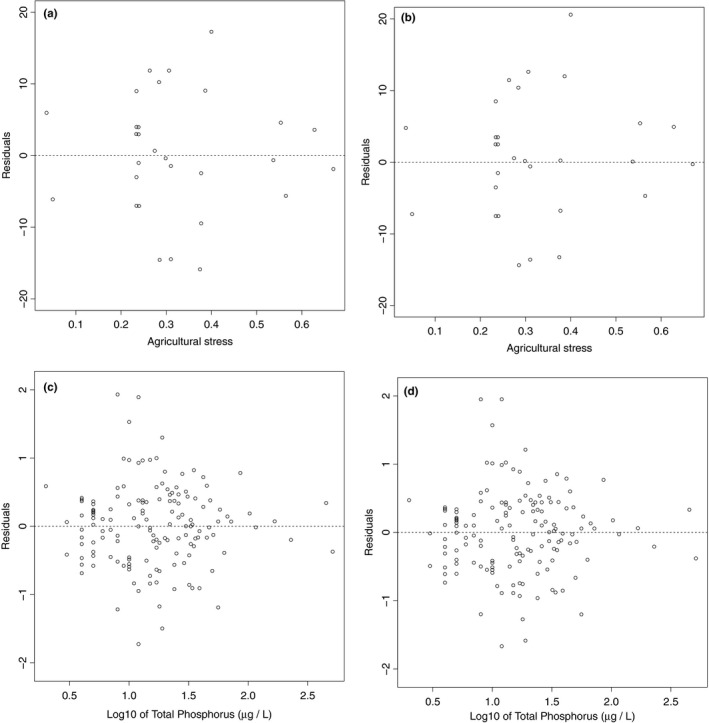
Plots of residuals for piecewise linear regression model (PLRM) and piecewise linear quantile regression model (PQRM) fitted to the fish multimetric index (Fish MMI) versus agricultural stress (AG) and cyanobacteria biomass (CB) versus total phosphorus (TP) data. In each subfigure, the residual standard deviation is denoted by σ. (a) PLRM to Fish MMI versus AG data with $\hat{\sigma }=9.055$, (b) PQRM to Fish MMI versus AG data with $\hat{\sigma }=9.179$, (c) PLRM to CB versus TP data with $\hat{\sigma }=0.614$, (d) PQRM to CB versus TP data with $\hat{\sigma }=0.618$

Following Qian (2014), we investigated the goodness‐of‐fit of our models by inspecting residual plots against environmental stress gradient (Figure A1). The residuals are distributed symmetrically around the horizontal line at 0 suggesting an absence of bias in the selected models. Again, the larger variability of residuals evident across the center of the environmental gradient than the edges supports the validity of using quantile regression.

Cade et al. (1999) showed the applications of linear quantile regression with varying error variances to two ecological applications and pointed out that estimating a range of regression quantiles provides a comprehensive description of biological response patterns for exploratory and inferential analyses. Cade and Noon (2003) explored the applications of both linear and nonlinear quantile regression models and showed how stronger and more useful predictive relationships can be found in other parts of the response distribution than that are observed only in the center. Cade et al. (2005) used linear and nonlinear quantile regression models to habitat data and showed that these models are less biased and uncertain than the classical models defined at the center. Brenden et al. (2008) used a collection of ecological models including the quantile piecewise linear (QPL) with applications in aquatic resource management to model a single breakpoint. Their models were mainly nonparametric in nature and depended heavily on CART. Moreover, the piecewise models were discontinuous in the threshold location between the two linear regimes and did not estimate the precision of the breakpoint. Feng et al. (2011) proposed wild bootstrap for linear quantile regression model via bootstrapping the residuals. In this paper, we have proposed a piecewise linear quantile regression model to detect two thresholds with continuous transition from one linear regime to the adjacent regime. We used wild bootstrap to identify the confidence intervals for the breakpoints and applied the proposed methods to two ecological datasets. The quantile regression estimates for the thresholds are less biased and more accurate than their counterparts of classical piecewise linear regression. Furthermore, the piecewise linear quantile regression model provides the smallest width of the prediction band for the simulated and real data especially for small samples and large error variances.

## CONFLICT OF INTEREST

None declared.

## AUTHOR CONTRIBUTIONS

**Jabed H. Tomal:** Conceptualization (equal); data curation (equal); formal analysis (lead); investigation (lead); methodology (lead); project administration (lead); resources (lead); software (lead); validation (lead); visualization (lead); writing–original draft (lead); writing–review and editing (lead). **Jan J. H. Ciborowski:** Conceptualization (equal); data curation (equal); funding acquisition (lead); investigation (equal); methodology (supporting); project administration (supporting); resources (supporting); supervision (lead); validation (supporting); visualization (supporting); writing–review and editing (supporting).

## Data Availability

The datasets used in this paper are available in the Dryad data repository and can be accessed via the following link (https://doi.org/10.5061/dryad.g79cnp5nr).

## Appendix A

**Table A1:** Means and 95% CIs of the ecological variables for the Fish MMI versus AG data and log(CB) versus log(TP) data

Source	Variables	n	Average	95% CI	
				Lower	Upper
Fish MMI versus AG					
GLEI^a^ & Uzarski^b^	Fish MMI	30	44.40	39.47	49.33
	AG		0.32	0.27	0.38
CB versus TP					
Alberta	CB	43	2.86	2.62	3.10
	TP		1.54	1.44	1.63
BC	CB	10	2.40	1.74	3.06
	TP		1.12	0.81	1.42
Ontario	CB	97	1.76	1.58	1.94
	TP		1.06	0.98	1.14
Combined	CB	150	2.12	1.96	2.28
	TP		1.20	1.13	1.27

^a^ Great Lakes Environmental Indicators (GLEI) by Bhagat et al. (2007).

^b^ Uzarski et al. (2005).

**Table A2:** Estimates, standard errors, t‐values, p‐values and 95% CIs of the thresholds and slopes of the PLRM lines fitted to the Fish MMI versus AG data

Names	Parameters	Fish MMI versus AG					
		Estimates	*SE*	t	p	95% CI	
						Lower	Upper
Thresholds	$\alpha_{1}$	0.263	0.033	7.970	0.000	0.196	0.331
	$\alpha_{2}$	0.488	0.047	10.383	0.000	0.391	0.585
Slopes	$\beta_{1}$	4.405	36.100	0.122	0.904	−70.110	78.920
	$\beta_{1}+\beta_{2}$	**−161.700**	53.810	−3.005	0.006	−272.800	−50.670
	$\beta_{1}+\beta_{2}+\beta_{3}$	112.300	80.680	1.392	0.177	−54.240	278.800

Note

The statistically significant slopes are highlighted using boldface.

**Table A3:** Estimates from the quantile regression at the median (τ=0.50) and 95% quantile‐based bootstrap confidence intervals of the thresholds and slopes of the piecewise linear quantile regression lines fitted to the fish MMI versus AG data

Names	Parameters	Fish MMI versus AG stress		
		Estimates	95% Confidence interval	
		Median (τ=0.50)	Lower	Upper
Thresholds	$\alpha_{1}\left(\tau \right)$	0.264	0.179	0.347
	$\alpha_{2}\left(\tau \right)$	0.466	0.393	0.553
Slopes	$\beta_{1}\left(\tau \right)$	1.447	−41.363	92.828
	$\beta_{1}\left(\tau \right)+\beta_{2}\left(\tau \right)$	−191.537	−410.675	−107.144
	$\beta_{1}\left(\tau \right)+\beta_{2}\left(\tau \right)+\beta_{3}\left(\tau \right)$	100.594	28.830	193.894

**Table A4:** Estimates of the thresholds ($\alpha_{1}\left(\tau \right)$ and $\alpha_{2}\left(\tau \right)$) and three slopes of the piecewise linear quantile regression models for different quantiles τ applied to the fish MMI versus AG data

Quantiles	Thresholds		Slopes		
τ	$\alpha_{1}\left(\tau \right)$	$\alpha_{2}\left(\tau \right)$	$\beta_{1}\left(\tau \right)$	$\beta_{1}\left(\tau \right)+\beta_{2}\left(\tau \right)$	$\beta_{1}\left(\tau \right)+\beta_{2}\left(\tau \right)+\beta_{3}\left(\tau \right)$
0.10	0.233	0.452	−3.054	−188.587	139.315
0.20	0.239	0.450	0.295	−199.208	139.736
0.30	0.233	0.449	29.514	−176.737	104.842
0.40	0.270	0.448	31.579	−223.464	88.891
0.50	0.264	0.466	1.447	−191.537	100.594
0.60	0.255	0.476	−10.076	−194.742	153.677
0.70	0.264	0.533	−1.939	−109.495	96.979
0.80	0.284	0.564	1.952	−106.214	120.274
0.90	0.264	0.551	23.924	−110.634	62.053

**Table A5:** Estimates, standard errors, t‐value, p‐value, and 95% CIs of the thresholds and slopes of the PLRM fitted to the CB versus TP data

Names	Parameters	CB versus TP					
		Estimates	*SE*	t	p	95% CI	
						Lower	Upper
Thresholds	$\alpha_{1}$	1.212	0.094	12.894	0.000	1.026	1.399
	$\alpha_{2}$	1.624	0.104	15.615	0.000	1.418	1.830
Slopes	$\beta_{1}$	**1.172**	0.326	3.595	0.000	0.528	1.815
	$\beta_{1}+\beta_{2}$	**3.344**	0.698	4.791	0.000	1.964	4.725
	$\beta_{1}+\beta_{2}+\beta_{3}$	0.831	0.441	1.884	0.062	−0.041	1.702

Note

The statistically significant slopes are highlighted by using boldface.

**Table A6:** Estimates from the quantile regression at the median (τ=0.50) and 95% quantile‐based bootstrap confidence intervals of the thresholds and slopes of the piecewise linear quantile regression lines fitted to the cyanobacteria biomass versus total phosphorus data

Names	Parameters	Cyanobacteria biomass versus total phosphorus		
		Estimates	95% Confidence interval	
		Median (τ=0.50)	Lower	Upper
Thresholds	$\alpha_{1}\left(\tau \right)$	1.110	0.829	1.408
	$\alpha_{2}\left(\tau \right)$	1.662	1.437	1.836
Slopes	$\beta_{1}\left(\tau \right)$	0.952	−0.316	1.816
	$\beta_{1}\left(\tau \right)+\beta_{2}\left(\tau \right)$	2.904	2.121	5.373
	$\beta_{1}\left(\tau \right)+\beta_{2}\left(\tau \right)+\beta_{3}\left(\tau \right)$	0.825	0.214	1.337

**Table A7:** Estimates of the thresholds ($\alpha_{1}\left(\tau \right)$ and $\alpha_{2}\left(\tau \right)$) and three slopes of the piecewise linear quantile regression models for different quantiles τ applied to the cyanobacteria biomass versus total phosphorus data

Quantiles	Thresholds		Slopes		
τ	$\alpha_{1}\left(\tau \right)$	$\alpha_{2}\left(\tau \right)$	$\beta_{1}\left(\tau \right)$	$\beta_{1}\left(\tau \right)+\beta_{2}\left(\tau \right)$	$\beta_{1}\left(\tau \right)+\beta_{2}\left(\tau \right)+\beta_{3}\left(\tau \right)$
0.10	1.228	1.609	0.509	3.564	1.269
0.20	1.201	1.585	0.562	4.414	0.853
0.30	1.066	1.645	0.364	3.532	0.724
0.40	1.230	1.646	1.072	3.693	0.688
0.50	1.110	1.662	0.952	2.904	0.825
0.60	1.176	1.641	1.342	2.931	0.758
0.70	1.155	1.623	1.565	2.679	0.794
0.80	1.146	1.598	1.541	2.874	0.647
0.90	1.200	1.623	1.958	2.946	0.689

**Table A8:** Comparison of width of the 95% CIs of the thresholds for the PLRM and PQRM. The top and bottom portions belong to the Fish MMI versus AG data and CB versus TP data, respectively

Methods	PLRM			PQRM		
Fish MMI versus AG						
Thresholds	95% CIs			95% CIs		
	Lower	Upper	Width	Lower	Upper	Width
$\alpha_{1}$	0.196	0.331	**0.135**	0.179	0.347	0.168
$\alpha_{2}$	0.391	0.585	0.194	0.393	0.553	**0.160**
CB versus TP						
$\alpha_{1}$	1.026	1.399	**0.373**	0.829	1.408	0.579
$\alpha_{2}$	1.418	1.830	0.412	1.437	1.836	**0.399**

Note

The smaller width in each row is highlighted by using boldface.

**Table A9:** Comparison of the surface areas in square units of the 80% and 95% prediction bands for loess, PLRM and PQRM

Fish MMI versus AG				
Methods	Confidence levels	Loess	PLRM	PQRM
Surface area	80%	**12.089**	17.240	13.668
	95%	**17.730**	27.000	22.078
CB versus TP				
Surface area	80%	**3.445**	3.956	3.485
	95%	**5.948**	6.073	6.099

Note

The top portion belongs to the Fish MMI versus AG data and the bottom portion belongs to the CB versus TP data. The smallest (most precise) surface area estimates are highlighted using boldface.
